# Strengthening stroke registries for trial readiness and research equity in Sub-Saharan Africa

**DOI:** 10.1016/j.ghrp.2026.09.001

**Published:** 2026-10-01

**Authors:** Benedict Kusi Ampofo, Fred Stephen Sarfo

**Affiliations:** a Directorate of Internal Medicine, Komfo Anokye Teaching Hospital, Kumasi, Ghana; b Department of Medicine, Kwame Nkrumah University of Science and Technology, Kumasi, Ghana

**Keywords:** Stroke, Registries, Sub-Saharan Africa, Health equity, Clinical trials

## Abstract

Sub-Saharan Africa carries one of the highest stroke burden globally, yet contributes fewer than 6% of participants to trials to inform international secondary stroke prevention guidelines. Although stroke registries across the region have expanded in numbers, they have not yet provided the trial-ready infrastructure required to support clinical research, quality improvement, and policy-relevant evidence generation. This commentary examines how existing stroke registries can evolve into platforms capable of supporting locally relevant clinical research and participation in multinational clinical studies. Recent studies identified more than 40 stroke registries across sub-Saharan Africa; however, most are characterised by heterogeneous data elements, variable follow-up, limited alignment with international standards, and incomplete population coverage. These structural limitations restrict comparability across sites, impede regional data pooling, and constrain participation in collaborative research. We propose a three-pillar pathway toward trial readiness comprising harmonised core data standards, operational capacity development through regional mentorship networks, and sustainable funding supported by coordinated national and international leadership. Harmonisation should be accompanied by broader registry coverage to improve representativeness and trial recruitment. Beyond supporting research participation, mature registries can also facilitate quality improvement, benchmarking, and health-system planning alongside clinical research. Transforming stroke registries into trial-ready platforms is both feasible and necessary to strengthen secondary prevention, improve research equity, and ensure that international stroke guidelines better reflect the epidemiological realities and research priorities of sub-Saharan Africa.

## Introduction

Stroke is the second leading cause of mortality worldwide.^1^ In some sub-Saharan African populations, age-standardised stroke incidence has been reported at approximately 316 per 100,000 population—among the highest observed globally.^2^ Despite this substantial burden, sub-Saharan Africa (SSA) remains critically underrepresented in the evidence base guiding stroke prevention and treatment. A recent analysis of 320 randomised controlled trials cited in major secondary stroke prevention guidelines found that fewer than 6% included participants from SSA, with all such participants exclusively recruited from South Africa.^3^

This disparity has tangible clinical consequences. Evidence from INTERSTROKE and the Stroke Investigative Research and Education Network (SIREN) studies indicates that stroke risk profiles in SSA differ substantially from those in high-income settings, with hypertension contributing a disproportionately greater share of stroke risk, a higher proportion of intracerebral haemorrhage, and stroke occurring at younger ages.^4^^,^^5^ Consequently, extrapolation of treatment effects from trials conducted predominantly in high-income settings to African populations should be interpreted cautiously, as differences in risk profiles and health-system contexts may affect guideline applicability in SSA.

A recent scoping review by Youkee et al. identified 42 stroke registries across 19 countries in sub-Saharan Africa and highlighted substantial heterogeneity in registry design, limited standardisation of data elements, variable reporting of follow-up, and inconsistent documentation of funding and governance structures.^6^ These findings raise a more fundamental policy question: can African stroke registries evolve from surveillance tools that merely document disease patterns into trial-ready infrastructure capable of contributing to the global evidence base informing secondary stroke prevention guidelines?

Building trial-ready registry infrastructure across SSA requires three essential components: data harmonisation, operational capacity development, and sustainable funding, each dependent on coordinated action by relevant stakeholders. Fig. 1 illustrates the proposed transformation of stroke registries in SSA from fragmented registry initiatives to trial-ready research infrastructure, highlighting key structural gaps and the enabling processes required for participation in guideline-generating trials.

**Fig. 1:**
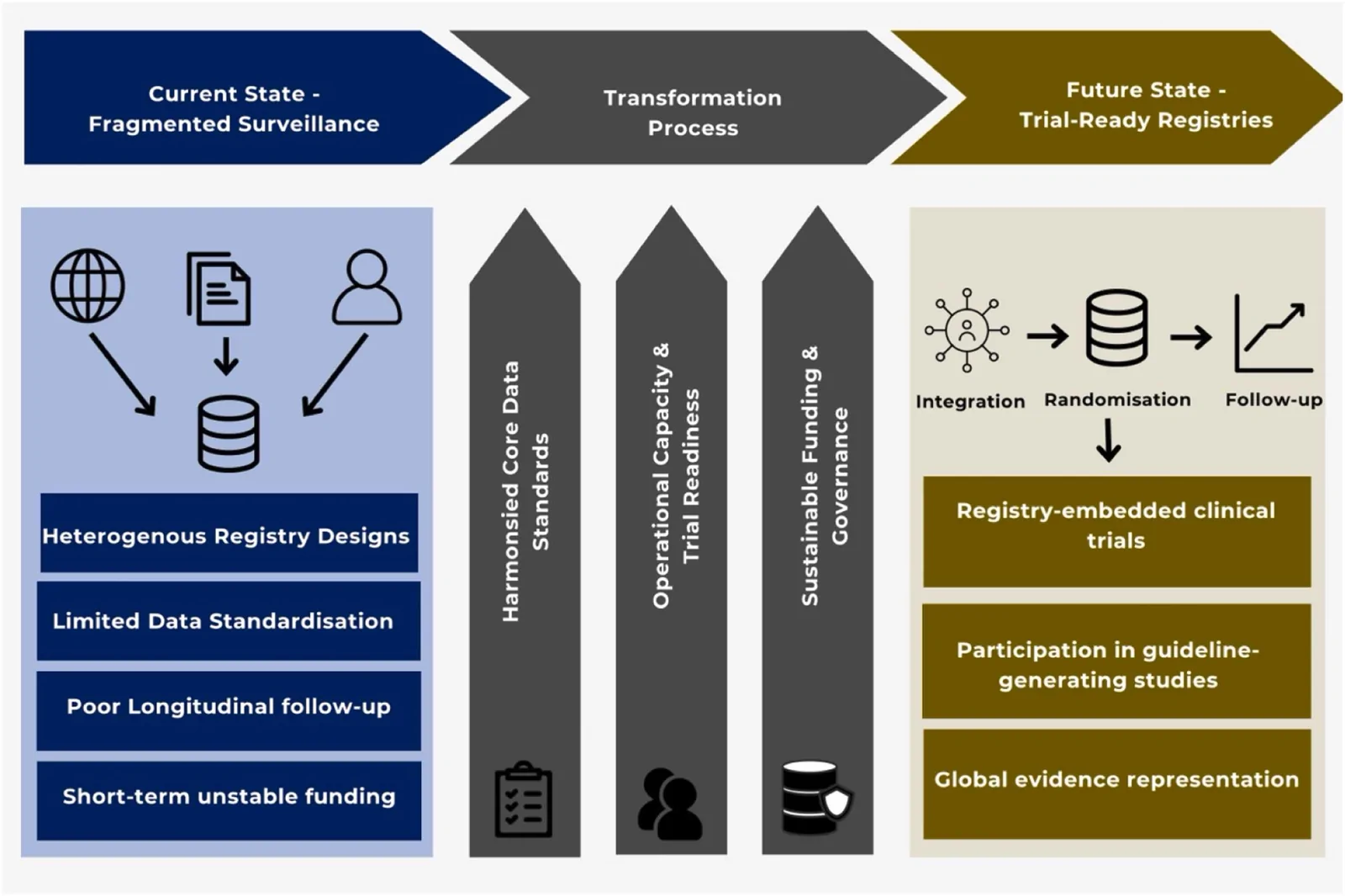
Conceptual framework illustrating the transformation of stroke registries in sub-Saharan Africa from fragmented registry initiatives to trial-ready research infrastructure. The figure highlights key limitations of current registries, the enabling processes required for transformation, and the resulting capacity for registry-embedded trials and participation in guideline-generating research.

## The case for data harmonisation

Stroke registries in SSA are highly fragmented due to uncoordinated design, with at least 42 registries identified across 19 countries and substantial variation in scope and data definitions.^6^ Only a minority apply internationally recognised standards for data collection, markedly limiting comparability across sites and countries.^6^ This heterogeneity constrains meaningful data pooling, benchmarking, and the generation of comparable regional estimates, thereby limiting participation in coordinated multi-country studies and randomised trials.

Previous global harmonisation initiatives, including WHO STEPwise surveillance and World Stroke Organisation registry recommendations, have had limited uptake across SSA due to fragmented health information systems, lack of sustained financing, and insufficient implementation support.^7^ In many settings, data standardisation is weakly linked to research participation or health policy use, limiting its perceived value and sustained uptake.^7^

Harmonisation does not require absolute uniformity; rather, it requires consensus on a minimal core dataset—drawn from established WHO and World Stroke Organisation frameworks—sufficient for trial eligibility screening, outcome assessment, and cross-site comparability. Future harmonisation efforts could be coordinated through collaboration between national governments, the World Stroke Organization, the World Health Organization, and regional professional societies. To sustain uptake, data standardisation should be linked to incentives for research participation and policy use, with harmonised registries prioritised for trials, guideline development, and system-level decision-making. Beyond supporting research participation, harmonised stroke registries can facilitate benchmarking, quality improvement initiatives, and monitoring adherence to evidence-based stroke care, similar to the role played by the Sentinel Stroke National Audit Programme (SSNAP) in the United Kingdom.^8^ However, harmonisation alone is insufficient if registries capture only a limited proportion of the stroke population. Expanding recruitment networks, strengthening case ascertainment, and increasing participation from secondary-level facilities may improve both representativeness and trial recruitment capacity.

Resource inequities across the region remain a major barrier to data harmonisation. Many centres lack continuous access to neuroimaging and face challenges with longitudinal follow-up. Well-designed harmonised registries can nonetheless capture these constraints systematically, thereby converting infrastructural limitations into evidence that informs health system planning and investment.

In practice, alignment with shared data standards enables harmonised registries to support registry-embedded trials that integrate randomisation and follow-up into routine clinical workflows. Sweden’s national stroke registry, which captures over 90% of stroke admissions using standardised variables, has supported multiple embedded randomised trials over the past decade.^9^ While developed in a high-income context, this illustrates the potential of standardised registries as trial platforms. With comparable data structures, SSA registries could adopt similar registry-based trial approaches, minimising recruitment and follow-up costs while strengthening African representation in guideline-defining stroke trials.

## Building operational capacity through regional networks

High-quality prospective stroke registries share several operational features with clinical trials, including standardised data collection, trained personnel, rigorous case ascertainment, and structured follow-up. However, participation in clinical trials additionally requires systems for eligibility screening, protocol implementation, safety reporting, trial governance, and adherence to Good Clinical Practice standards. Bridging the gap between conventional registry functions and trial readiness therefore requires deliberate investment in operational infrastructure, workforce development, governance, and quality assurance.

Successful models for building such research capacity already exist across Africa. The Human Heredity and Health in Africa (H3Africa) Consortium established sustained multicountry research networks that combined shared infrastructure, coordinated governance, harmonised protocols, and targeted training to strengthen research capacity across diverse settings.^10^ Through its collaborative framework, H3Africa demonstrated that regional mentorship and coordinated infrastructure can support participation in complex multicentre research despite resource constraints.^10^ Similar regional approaches could be adapted to strengthen stroke registries, enabling their evolution into trial-ready platforms capable of supporting participant recruitment, protocol delivery, safety monitoring, and high-quality clinical research.

Applying similar principles to stroke registries would support the development of sites capable of conducting high-quality observational studies while progressively building the infrastructure required for future registry-embedded clinical trials. Translating this vision into practice will require targeted policy action. Regional centres of excellence should receive dedicated funding to mentor emerging sites in protocol implementation, research ethics, data quality assurance, and trial coordination. Electronic data capture platforms should be designed for intermittent connectivity, while research coordinator certification programmes could promote consistent operational standards across participating centres. Such investments have multiplier effects, enabling experienced sites to strengthen capacity across regional research networks progressively.

## Reorienting funding toward sustainability

The scoping review by Youkee et al. highlights limited evidence of long-term registry continuity, while broader analyses of African health research systems demonstrate that overreliance on short-term and donor-driven funding undermines registry sustainability.^6^^,^^11^ Recent reviews of donor-funded health interventions show that many programmes do not persist beyond the funding lifecycle, with external funding volatility and weak domestic investment limiting long-term capacity.^11^ These constraints limit staff retention, continuity of data capture, and longitudinal follow-up, hindering the maturation of registries into platforms capable of supporting long-term research, quality improvement, and clinical trials. These repeated cycles dissipate prior investments and prevent registries from achieving the operational maturity required to support longitudinal research and trials.

Performance-based funding can promote sustainability by linking financial support to data quality and meaningful research participation. Core operational support should be contingent on verified data completeness and periodic external quality audits, with additional funding linked to meaningful research contributions, including enrolment of participants into approved clinical trials. For example, registries achieving 90% data completeness and participating in at least one approved trial annually could qualify for enhanced funding tiers. These incentive structures must reward demonstrable data quality and collaborative research participation.

Achieving sustainability will require coordinated investment by national governments and international funders, with stroke registries supported as core public health infrastructure in line with World Health Organisation targets and funded beyond short-term and project-bound cycles. Funders should earmark a defined proportion of grant portfolios specifically for long-term registry sustainability, rather than limiting support to short-term and project-bound data collection.

The return on this investment is substantial. Well-maintained registries support benchmarking, quality improvement, health-system planning, and the generation of real-world evidence for policy and regulatory decision-making. In addition, they provide essential infrastructure for participation in registry-embedded multinational clinical trials that contribute to the evidence base underpinning international stroke guidelines.

## Conclusions

Stroke registries in SSA have successfully documented disease burden but remain insufficiently equipped to generate the evidence required to inform clinical guidelines. Without deliberate investment in harmonisation, trial readiness, and long-term sustainability, African populations will remain underrepresented in the trials that define global stroke care. Until African populations are adequately represented in guideline-generating trials, global stroke recommendations will remain incompletely generalisable, thereby perpetuating inequities in both evidence generation and stroke outcomes across the continent.

## List of Abbreviations

H3Africa: Human Heredity and Health in Africa
LMIC(s): Low- and Middle-Income Country(ies)
SIREN: Stroke Investigative Research and Education Network
SSA: Sub-Saharan Africa
WHO: World Health Organisation
WSO: World Stroke Organisation

## CRediT authorship contribution statement

**Benedict Kusi Ampofo:** Writing – review & editing, Writing – original draft, Visualization, Conceptualization. **Fred Stephen Sarfo:** Writing – review & editing, Supervision.

## Ethics approval and consent to participate

Not applicable.

## Consent for publication

Not applicable.

## Funding

None.

## Declaration of Generative AI and AI-assisted technologies in the writing process

Artificial intelligence–assisted tools were used for language refinement and clarity. All authors take full responsibility for the content of the manuscript.

## Declaration of Competing Interest

None to declare.

## Data Availability

No data was used for the research described in the article.

## References

[1] Feigin V.L., Stark B.A., Johnson C.O. (2021). Global, regional, and national burden of stroke and its risk factors, 1990–2019: a systematic analysis for the Global Burden of Disease Study 2019. Lancet Neurol.

[2] Owolabi M.O., Akarolo-Anthony S., Akinyemi R. (2015). The burden of stroke in Africa: A glance at the present and a glimpse into the future. Cardiovasc J Afr.

[3] Opare-Addo P.A., Sarfo F.S., Berchie P.O., Aikins M., Ovbiagele B. (2023). Participation by patients from low- and middle-income countries (LMICs) in trial evidence supporting secondary stroke prevention guideline recommendations. J Neurol Sci.

[4] O’Donnell M.J., Chin S.L., Rangarajan S. (2016). Global and regional effects of potentially modifiable risk factors associated with acute stroke in 32 countries (INTERSTROKE): a case-control study. Lancet.

[5] Owolabi M.O., Sarfo F., Akinyemi R. (2018). Dominant modifiable risk factors for stroke in Ghana and Nigeria (SIREN): a case-control study. Lancet Glob Heal.

[6] Youkee D., Baldeh M., Rudd A. (2025). A scoping review of stroke registers in Sub-Saharan Africa. Int J Stroke.

[7] Riley L., Guthold R., Cowan M. (2016). The World Health Organization STEPwise approach to noncommunicable disease risk-factor surveillance: methods, challenges, and opportunities. Am J Public Health.

[8] Healthcare Quality Improvement Partnership. Sentinel Stroke National Audit Programme (SSNAP) 2026.

[9] Asplund K., Hulter Åsberg K., Appelros P. (2011). The Riks-Stroke story: building a sustainable national register for quality assessment of stroke care. Int J Stroke.

[10] H3Africa Consortium (2014). Research capacity. Enabling the genomic revolution in Africa. Science.

[11] Nwaozuru U., Murphy P., Richard A. (2025). The sustainability of health interventions implemented in Africa: an updated systematic review on evidence and future research perspectives. Implement Sci Commun.

